# Effects of care assistant communication style on communicative behaviours of residents with dementia: a systematic multiple case study

**DOI:** 10.1111/scs.12622

**Published:** 2018-10-17

**Authors:** Miriam Stanyon, Shirley Thomas, Adam Gordon, Amanda Griffiths

**Affiliations:** ^1^ Division of Psychiatry & Applied Psychology School of Medicine Institute of Mental Health University of Nottingham Nottingham UK; ^2^ Division of Rehabilitation & Aging School of Medicine University of Nottingham Nottingham UK

**Keywords:** case study, communication, dementia, experimental method, residential care

## Abstract

**Objectives:**

To determine whether varying the communication style of care assistants, encouraging them to use direct instructions and allowing more time for residents’ responses influenced the communicative behaviour of care home residents living with dementia.

**Design:**

This study used a multiple systematic case study design. Participants were video‐recorded during morning care routines in three communication conditions: usual communication, direct instructions and pacing (allowing more time for resident responses). Each dyad acted as its own control.

**Setting:**

The study took place in a residential care home in the East Midlands, UK.

**Participants:**

Three dyads (person with dementia/care worker)

**Measures:**

The level of compliance with instructions was measured. Validated measures were used to rate positive communicative behaviour (engagement with care tasks, eye contact and initiation of interaction) and negative communicative behaviour (e.g. shouting and kicking).

**Results:**

Care assistants were able to employ direct instructions after brief training. The use of direct instructions was positively correlated with positive communicative behaviour from residents (p < 0.05). The pacing condition was not employed adequately to evaluate effectiveness. Negative communicative behaviour (resistiveness to care) was rare.

**Conclusion:**

The use of direct instructions by care assistants holds promise for effective communication with people with dementia and warrants further investigation in larger samples and in varied contexts.

## Introduction

Communication impairments are among the earliest symptoms of dementia. Word‐finding and comprehension problems can contribute to disorientation, anxiety and behavioural symptoms such as resistance to care or withdrawal from social interaction 1, 2. Approximately a third of people with dementia (PwD) in the UK live in care homes 3. Effective communication is crucial in establishing the carer/care recipient relationship which has been found to contribute to the meeting of care needs and improvements in behaviour and well‐being in care recipients 4, 5 and burnout rates in care assistants 6. Care assistants need to be able to use communication with the optimum characteristics to understand the needs of PwD and to communicate their intentions as clearly as possible 7. Effective communication is a key factor in the successful completion of activities of daily living (ADLs) 8, 9, and many care assistants use ADLs as an opportunity to spend social time with the residents they care for 10, 11. Improving communication between care assistants and PwD during ADLs is therefore likely to be an effective way to maximise existing communication opportunities.

Person‐centred care is generally accepted to be the approach of best practice in dementia care and holds to the principle of therapeutic communication between care assistant and a PwD 12. Kitwood explains that, rather like a skilled tennis coach playing a rally with a less skilled player, a care assistant must use their communication skills and resources to facilitate the interpretation of meaning and shared understanding. It is therefore imperative that care assistants are trained in this therapeutic approach and have a body of skills to draw upon when conversing with a PwD.

There is a growing body of literature regarding training care assistants in communication skills 7, 10, but current training programmes are based predominantly upon clinical experience and little work has been undertaken to experimentally establish the effective components of communication 13, 14, 15. An empirical evidence base is required in order to construct a gold standard method of communication. Literature on communication between care assistants and PwD has suggested that certain speech characteristics such as elderspeak 16, 17, 18, 19, a style of speech used by younger people when addressing older people, or a controlling vocal tone 20 may cause PwD to resist essential care activities. In an earlier study with health and social care staff who communicate with PwD regularly, a list of potentially helpful communication strategies was generated which included a variety of verbal and nonverbal techniques, as well as personal and organisational factors 11. Of these, two strategies were thought to be amenable to experimental manipulation: direct instructions and pacing.

Direct instructions are defined as short instructions which are precise rather than vague and possible for the recipient to complete. Such instructions have been described in the literature as ‘alpha commands’. It has been shown experimentally that ‘alpha commands’, for example ‘roll over to the right’, are associated with greater compliance than more vague ‘beta commands’, for example ‘move over’ 21. Complexity of syntax has also been shown to affect comprehension in PwD 22, 23. Direct instructions only include one instruction in the sentence rather than multiple instructions such as ‘Roll over and stay there for me while I wash and dry your back’, often referred to as compound instructions.

Pacing involves slowing the speed of turn‐taking in an interaction, allowing more opportunity for a response from the conversation partner. This does not refer to the slowing of speech rate but the augmenting of the time given to the conversation partner to consider and offer a response. In a small intervention study, teaching about pacing was part of a successful multifaceted staff training intervention 15 where residents in the treatment group displayed higher coherence of speech and a lower occurrence of empty phrases compared to the control group postintervention. However, it is not known whether pacing was one of the active ingredients that contributed to its success. Also unknown is whether the care assistants employed pacing as intended.

This study aimed to determine whether communication between care assistants and PwD could be experimentally manipulated through use of direct instructions and pacing and whether doing so had a measurable impact upon PwD's positive communicative behaviour and resistiveness to care.

## Methods

### Design

This study employed a multiple systematic case study design 24 where each participant served as his or her own control. Three care worker–resident dyads were video‐recorded on four separate occasions while completing the morning care routines where the PwD was assisted to wash and dress. The washing and dressing activity was chosen as it is an ADL where the care assistant typically gives numerous instructions and so would give ample opportunity for the communication strategies to be used. Morning care is also generally a one‐to‐one activity and takes place in the same environment every day, reducing the possible introduction of confounding variables. Morning care also takes place over the period of 15–30 minutes allowing for multiple data collection points. Care assistants employed different communication strategies on separate days in an A^1^BA^2^C design where A^1^ and A^2^ represented communication as usual, B the use of direct instructions and C the use of pacing. By asking care assistants to return to their usual communication style between conditions, this would allow a ‘washout period’ so that the effects of the direct instructions intervention would not impact any response measured in the pacing condition. In the direct instructions condition, the care assistants were asked to use short, precise instructions and to avoid using compound instructions, where more than one instruction is given in one sentence, instructions phrased as a question, for example ‘Can you lift your hands for me?’ 21, or syntactically complex sentences. In the pacing condition, the care assistants were asked to leave at least 5‐second from the end of their instruction before either repeating the instruction or initiating a new action.

### Setting

All data collection occurred in a privately owned, residential care home located in the East Midlands of the UK. Of the four care facilities invited to participate, this was the only facility to accept. The facility was not a dementia specialist home and catered for fewer than 50 residents with a variety of physical and cognitive impairments. Approximately 60% of the residents had a diagnosis of dementia or cognitive impairment. The staff‐to‐resident ratio per shift was approximately 1 : 5. Video recording took place in residents’ bedrooms or en suite bathrooms where their morning care routine normally took place. All recording occurred between 6:30 am and 10:00 am at the time when each resident would routinely be woken and receive assistance with washing and dressing. All data collection took place between April and July 2013.

### Participants

Participants consisted of three care assistants paired with three care home residents with dementia. These dyads remained constant throughout all conditions. Participant characteristics are summarised in Table 1.

**Table 1:** Participant characteristics

Care assistants					
Dyad	Gender	Age	Ethnicity	Experience with dementia	Experience with resident
1	Male	28	White British	7 years	3 months
2	Female	53	White British	4 years	4 years
3	Male	19	White British	8 months	3 months

Residents with dementia						
Dyad	Gender	Age	Ethnicity	Diagnosis	MMSE score	Time in care home
1	Female	84	White British	AD	6/30	3 months
2	Female	92	White British	VaD	1/30	6 years
3	Female	85	White British	AD	13/30	6 months

AD, Alzheimer's disease; VaD, vascular dementia.

Care assistants were recruited first through their manager who was asked to identify care assistants who had been employed at the home for over 6 months and were English speaking. Care assistants who expressed an interest were given information sheets where they were informed of their right to withdraw at any time. They were given the opportunity to ask any questions and then asked to sign a consent form. Of the six care assistants who were given information about the study, three consented. The care assistants were asked to identify residents in their care who fulfilled the following inclusion criteria: English as their first language, a diagnosis of dementia or ‘probable dementia’, sufficient auditory and visual acuity to take part in interaction and a requirement for assistance/supervision with ADLs. Potential participants were asked by the care assistant if they were willing to talk to the researcher. If they were, the researcher informed them about the research study and, at this time, assessed their mental capacity to give informed consent to be involved in the research. None of the prospective resident participants had the mental capacity to give informed consent, and consultees were found in accordance with the Mental Capacity Act 2005. Written consent was given by the PwD and their consultees after a joint conversation with the researcher. Recruitment took place in March and April 2013.

### Data collection

Before morning care began, the researcher reminded the resident about the research and the video recording due to take place. The video camera was placed in an appropriate position to film both the resident and the care assistant during morning care and recording began before the researcher left the room. The camera was retrieved once the care routine was completed. The film was edited, to delete sections where residents were undressed, before analysis in accordance with the conditions of the research ethics committee.

Eighteen morning care routines were filmed, six for each dyad. Two pilot sessions were filmed to enable acclimatisation of participants to the presence of the video camera and adjustments to the camera position. Data from the pilot sessions were not used in the analysis. Following these two sessions, the researcher recorded one session for baseline communication as usual, direct instructions, return to communication as usual and pacing. In between recording days, the care assistants were asked to continue communication as usual. On average, a week would pass between recorded sessions.

Training sessions took place between communication as usual conditions and the commencement of the direct instructions and pacing conditions. Training was delivered to care assistants individually and lasted 20 minutes. Training consisted of a short presentation by the researcher informing the care assistant of the communication strategy to be employed. The care assistant was then given the opportunity to practice the strategy in a series of exercises involving role‐play. The researcher gave feedback and used a structured question and answer session to reinforce learning. Carers were given a brief 10‐minute refresher session directly before filming and asked not to discuss the content of the training with others to prevent contamination.

### Measures

Care assistants’ fidelity in the direct instructions condition was measured by categorising all instructions as either direct or nondirect according to definitions used in previous research 21. The percentage of direct instructions as a proportion of all instructions was captured for all conditions to investigate the extent to which care assistants increased their use of direct instructions after training. Fidelity in the pacing condition was gauged by measuring the time between the end of the instruction and the PwD's response, or between the end of the instruction and the initiation of a new action by the care assistant. The mean average of each time was calculated and compared with those in the usual communication conditions.

The PwD's positive communicative behaviour was rated using three subscales of the Positive Response Schedule for Severe Dementia (PRS) 25. This measures the frequency of three microbehaviours: looking at carer, initiating interaction and engagement. This measure was chosen for its ability to capture the microbehaviours of PwD over a short period of time without relying on caregiver report; the reliability of the latter has often been questioned in the literature 26. The interobserver reliability of the PRS has ranged from 80% to 99% in previous research 25, 27. Video recordings were divided into 20‐second intervals, and the presence or absence of microbehaviours in each interval was noted. This 20‐second time interval was initially used by the authors of the PRS so that an observer could observe for 20 seconds and then record their observations for 10 seconds. Due to the video recording of the interactions, this recording time was not necessary; however, these 20‐second intervals allowed the researcher to follow the fluctuation of positive or negative communicative behaviour in relation to the occurrence of care assistant actions over the course of the interaction. Summary PRS scores were calculated by summing the total score from each session.

The PwD's negative communicative behaviour was measured using the Resistiveness to Care Scale (RTC‐DAT) 28, 29, which measures gegenhalten (body movements of equal force but in the opposite direction from the caregiver); grabbing objects; saying ‘no’; adduction (clenching the limbs near the body); grabbing people; pulling away; clenching; crying; screaming; turning away; pushing away; hitting/kicking; and threatening. Each occurrence of resistive behaviour was rated by duration and intensity. Duration was rated on a five‐point scale: 0 (absent), 1 (<16 seconds), 2 (16–59 seconds), 3 (1–2 minutes) or 4 (>2 minutes). Intensity was rated on a three‐point scale: 1 (mild), 2 (moderate) or 3 (extreme). The duration and intensity scores were multiplied and summed to give a total score for the interaction. Again, this measure was chosen for its ability to capture the behaviour of a PwD over a short period of time without relying on caregiver report. Interobserver reliability for the RTC‐DAT has been reported at 95%, and construct validity was established with a principal component factor analysis reporting a three‐factor solution explaining 52.3% of variance 29.

In addition to validated scales, compliance was recorded. Compliance was defined as ‘appropriate behaviour initiated within 5‐second following an instruction that terminated with the completion of the assigned task’ 21 and noncompliance as ‘failure to initiate an appropriate response within 5‐second following an instruction issued by the care assistant’. Forced compliance was defined as ‘a requested response completed by the care assistant, instead of the resident, within 5‐second of the instruction’. Every instruction issued by the care assistants was coded as resulting in compliance, noncompliance or forced compliance.

The coding of 25% of the overall video data (20 minutes of video recordings) was validated by a second rater. Two minutes from each videoed session were chosen randomly, and the coding of a second rater was compared with the coding of the primary researcher. Kappa coefficient tests were carried out to determine interobserver reliability on command category, the PRS, the RTC‐DAT and measures of compliance. Agreement was very high for command type, compliance and RTC‐DAT (*k* = 0.80–0.81) and acceptable for the PRS (*k* = 0.61).

### Analysis

Video recordings were analysed using the ELAN video analysis tool (version 4.1.0, Max Planck Institute for Psycholinguistics). This is a software tool often used by linguists and allows video and auditory data to be slowed down to observe and make notes on the incidents and duration of microbehaviours. The length of the videos varied from 12 minutes and 24 seconds to 25 minutes and 11 seconds; therefore, summary scores represented as percentages and averages were used throughout the analysis process. Communication style, the instruction type, pacing scores, PRS, RTC‐DAT and compliance scores for each condition within each dyad were compared using chi‐squared tests as the assumptions of parametric testing were not met and this was the test recommended for use in the original PRS study 25. The PRS subscales were not analysed separately as this was not cogent to the research question being addressed in this study. Summary scores from each condition across dyads were compared to determine whether there was any relationship between care assistant communication style and PwD communicative behaviour using Spearman's Rho. Significance was set at p > 0.05.

## Results

### Care assistant adherence

Table 2 shows the percentages of direct instructions and the time lapse between instructions and the initiation of a new activity for each condition and dyad. After the direct instructions training, all care assistants increased the proportion of direct instructions from the proportion used in the baseline condition. In dyads 2 and 3, there were significant increases in the proportion of direct instructions used, though not always in the direct instructions condition. Two care assistants – those in dyads 1 and 3 – maintained higher levels of direct instructions in the pacing condition, despite being asked to return to their usual communication style.

**Table 2:** Percentages of direct instructions and average time for response from instructions for each condition in each dyad

Dyad	1				2				3			
Condition	Usual 1	Direct Instructions	Usual 2	Pacing	Usual 1	Direct Instructions	Usual 2	Pacing	Usual 1	Direct Instructions	Usual 2	Pacing
% Direct instructions	56.4	76.7	66.7	84.8	46.0	70.6^a^	32.3	80.0^a^	51.5	58.5	86.7^a^	65.5
Mean time lapse (sec)	1.2	1.0	2.1	3.6	0.1	0.6	0.0	−0.7	1.4	1.7	0.8	2.3
Range (sec)												
Min	−0.92	−1.76	−1.78	−2.36	−2.66	−2.49	−1.8	−1.7	−0.52	−0.56	−0.89	−0.71
Max	4.73	4.8	5.28	11.56	2.07	2.3	3.99	0.91	4.11	3.96	3.08	5.01

Significant difference of condition percentages and means within each dyad calculated using chi‐squared tests.

a p < 0.05.

In the pacing condition, the care assistants did not significantly increase the average time lapse between instructions and care assistant response.

### Resident behaviour

Positive Response Schedule scores differed significantly across conditions for all dyads. Resistiveness to care was rare throughout the study. Only dyad 2 showed enough resistive behaviour to be eligible for statistical analysis – but statistically significant differences were seen across conditions for this dyad with a significantly lower level of resistive behaviour apparent in the direct instructions condition. These results are summarised in Table 3.

**Table 3:** PRS scores and RTC‐DAT scores for each condition in each dyad

Dyad	1				2				3			
Condition	Usual 1	Direct Instructions	Usual 2	Pacing	Usual 1	Direct Instructions	Usual 2	Pacing	Usual 1	Direct Instructions	Usual 2	Pacing
PRS score	35.2	74.4^a^	63.3	61.9	56.3	70.8^b^	47.2	93.3^b^	47.3	31.4	69.7^a^	33.3
RTC‐DAT score	n/a	n/a	n/a	n/a	17.5	1.0^a^	8.3	4.7	n/a	n/a	n/a	41.0

Significant difference of condition scores within each dyad calculated using chi‐squared tests.

a p < 0.05.

b p < 0.005.

The rates of compliance were significantly higher and forced compliance significantly lower across all three dyads when direct instructions were compared with nondirect instructions. In all three dyads, the majority of nondirect instructions resulted in noncompliance or forced compliance and direct instructions resulted in an increased proportion of compliance to noncompliance as can be seen in Table 4.

**Table 4:** Rates of instruction type and compliance in each dyad across all conditions

Dyad	Instruction type	Compliance	Noncompliance/forced compliance	Totals	Percentage compliance
1	Direct instructions	56^a^	76	132	42.4
	Nondirect instructions	13	57	70	18.6
	Totals	69	133	202	34.2
2	Direct instructions	50^a^	16	66	75.8
	Nondirect instructions	22	70	92	23.9
	Totals	72	86	158	45.6
3	Direct instructions	29^a^	30	59	49.2
	Nondirect instructions	11	34	45	24.4
	Totals	40	64	104	38.5

Significant difference of condition percentages within each dyad calculated using chi‐squared tests.

a p < 0.001.

### Correlational analyses

Correlational analysis showed there to be a moderate positive association between PRS scores and the percentage of direct instructions; *r* = +0.65, p < 0.05. The correlation between direct instructions and RTC‐DAT scores was found to be insignificant; *r* = −0.32, p > 0.05. No correlation was found between direct instructions and rates of compliance; *r* = −0.07, p > 0.05.

## Discussion

This is the first study to experimentally manipulate the use of isolated communication techniques by care assistants of PwD in a naturalistic setting. Research to date has examined communication in a laboratory setting 18, 22, 23, 30, 31, has only observed behaviour in a naturalistic setting 16, 17, 20, 21 or have not looked at techniques in isolation 14, 15.

The main findings of this study were that the use of direct instructions by care assistants was correlated with an increase in the communicative behaviour of care home residents with dementia and greater compliance. Greater proportions of compliance were observed with a care assistant's use of direct instructions and greater proportions of noncompliance and forced compliance observed when a care assistant's instructions were nondirect. Dyads failed to use the pacing communication strategy adequately, and therefore conclusions cannot be drawn about the effect of this strategy on the communicative behaviour of PwD. It was also interesting to note that the direct instructions strategy may be difficult to unlearn as care assistants continued to use direct instructions in subsequent conditions despite a total of 2 weeks passing from the training to the filming of the pacing condition, and being asked not to do so.

These results provide empirical evidence to support the hypotheses in the literature that direct instructions, which are characterised by sentences which are short, syntactically simple and precise, are both easier to understand and lead to greater compliance in PwD 21, 22, 23, 32. This study adds to these findings that direct instructions can encourage people with dementia to show more positive communicative behaviour themselves, engaging with tasks with the care worker and even initiating interaction themselves. This increase in instances of PwD initiating interaction could indicate that the use of direct instructions may help prevent the withdrawal often displayed in PwD 31. These findings relate back to person‐centred care theory in that direct instructions, thought to facilitate the interpretation of meaning and shared understanding, seemed to encourage a greater number of positive communicative acts from the PwD. This shows that direct instructions could form one of the components of therapeutic communication as proposed by Kitwood 12.

The pacing strategy was not adequately administered due to care assistants often using an instruction, either direct or indirect, as an explanation of the care assistant's actions rather than a request for the PwD to act. For example, the care assistant may say ‘Can you lift your foot for me so I can put your sock on?’ simultaneously to lifting the PwDs foot herself. It may be that the training of the care assistants was of insufficient duration or frequency to allow care assistants to reflect adequately on their current practice and how certain of its characteristics should change when new strategies are employed. Another reason for the pacing condition not being adequately administered was due to the PwD often responding to instructions inappropriately quickly after the instruction. It may be that pacing would be more effective in certain subtypes of dementia such as dementia with Lewy bodies, which often includes symptoms such as Parkinsonism where the PwD takes an extended period of time to process and respond to an instruction.

The strengths of this study lie in the innovative use of video data to enable real‐time analysis of complex verbal and nonverbal interactions, which has been shown to be more reliable than retrospective reports from care assistants 13. These videos captured a richness of communication between care assistant and PwD which could be lost in other forms of data collection. Although there is a risk of response bias, previous studies have found, and this study can further support the claim, that the presence of a video camera is soon forgotten and participants begin to act as if the camera were not there 33. The author argues that this response bias would have been greater had the researcher been in the room and observing the interaction. A further strength was that the communication interventions were selected based upon a hypothesis driven by previous research undertaken with care assistants 32 and were therefore accessible and feasible when presented to the care assistants who were to employ them. The limitations of this study are the small number of participants and observations. This limits the generalisability of findings. In addition, these results were observed only in morning care situations. Communication may change in different situations, such as assisting a resident to the toilet or at mealtimes, or with different communication partners, such as in a triad with a family member. It may be possible that these situations would also capture a greater level of resistive behaviour. The initial findings from these small case studies should be tested in larger samples, with a greater number of observations and in situations outside of the morning care routine.

The results of this study suggest that further research should be carried out into the effect of direct instructions on the communicative behaviour of PwD. It would also be useful to see the longer term effects of improved communication in a care context. Direct instructions reliably result in greater compliance, and this may have implications for time‐saving and greater job satisfaction for care assistants. Further, because such instructions are easier to follow, they may reduce distress for PwD.

## Conclusion

In this small study, a positive relationship has been found between the use of direct instructions by care assistants and positive communicative behaviour of PwD. Their use warrants further study with a larger sample and in varied settings. The use of direct instructions has possible implications not only for care assistant effectiveness and job satisfaction but also for reducing distress and increasing positive communicative behaviour in care home residents with dementia.

## Conflict of interest

None.

## Author contributions

All authors have consented to this submission. All data were collected and analysed by Dr Miriam Stanyon. Project design and guidance throughout the project were given by Professor Amanda Griffiths, Dr Shirley Thomas and Dr Adam Gordon. The manuscript was written by Dr Miriam Stanyon and critically revised by Dr Adam Gordon, Professor Amanda Griffiths and Dr Shirley Thomas.

## Ethical approval

This study was granted ethical approval by the Ethics committee of the Institute of Work, Health and Organisations at the University of Nottingham. Written consent was obtained from all participants and their consultees.

## Funding

This work was supported by the Economic and Social Research Council [grant number ES/I021132/1] through a studentship awarded to the first author.
